# From Traumatic Brain Injury to Alzheimer’s Disease: Multilevel Biomechanical, Neurovascular, and Molecular Mechanisms with Emerging Therapeutic Directions

**DOI:** 10.3390/ijms27031570

**Published:** 2026-02-05

**Authors:** Aikaterini Katramadou, Eva Sonja Bender, Dimitrios Kanakis

**Affiliations:** 1 Laboratory of Pathology, Department of Basic and Clinical Sciences, University of Nicosia Medical School, Nicosia 2408, Cyprus; katramadou.a@live.unic.ac.cy (A.K.); bender.e@live.unic.ac.cy (E.S.B.); 2 Centre of Neuroscience and Integrative Brain Research (CENIBRE), University of Nicosia Medical School, Nicosia 2408, Cyprus

**Keywords:** traumatic brain injury (TBI), Alzheimer’s disease (AD), chronic traumatic encephalopathy (CTE), diffuse axonal injury (DAI), neurovascular changes, therapeutic interventions

## Abstract

Traumatic brain injury (TBI) is being increasingly recognized as a major risk factor for chronic neurodegenerative disease, including chronic traumatic encephalopathy (CTE) and Alzheimer’s disease (AD). Biomechanical forces during head trauma, particularly rotational acceleration and angular deformation, produce diffuse axonal injury (DAI) and widespread white matter damage that trigger persistent neurobiological cascades. These include axonal transport failure, blood–brain barrier (BBB) disruption, neuroinflammation, neurovascular and mitochondrial dysfunction, and pathological protein aggregation, closely paralleling core AD features. Epidemiological data support a dose–response relationship between TBI severity or repetition and subsequent dementia risk, moderated by genetic factors such as apolipoprotein E4 (ApoE4). Converging experimental and early clinical studies have begun to target shared injury and neurodegenerative pathways through acute neuroprotection, stem cell-based strategies for BBB restoration and neural repair, transcriptional and hormonal modulation, mitochondrial stabilization, and immunomodulation of chronic inflammation. This review synthesizes evidence linking biomechanical injury to molecular and neurovascular pathways of neurodegeneration and summarizes emerging temporally targeted interventions. By integrating mechanistic and therapeutic perspectives, we aim to narrow the translational gap between TBI and AD, refine identification of at-risk populations, and inform priorities for prevention and development of disease-modifying therapies.

## 1. Introduction

### 1.1. Traumatic Brain Injury (TBI): Definition, Prevalence, and Biomechanics

Traumatic brain injury (TBI) is a significant global public health concern substantially contributing to mortality and disability with major medical, social, and economic impacts. TBI results from an external force causing temporary or permanent cognitive, physical, or psychosocial impairment [1]. Mechanical energy is transferred to the head by events such as direct impact, penetrating injury, or rapid acceleration and deceleration of the brain within the skull. In 2021, there were 20.84 million new and 37.93 million existing TBI cases globally [2]. Estimates vary, but TBI is widely recognized as affecting over 50 million people annually and accounting for 30–40% of all injury-related deaths worldwide [3,4].

Epidemiological patterns indicate that TBI varies significantly based on geography, socioeconomic context, age, and sex. In low- and middle-income countries, road traffic collisions account for most cases. However, in higher-income countries, most TBIs are caused by falls, particularly in the older population with other comorbidities [3]. Sports-related concussions and military blast exposures notably contribute to TBI incidence [5]. In most settings, young males experience a disproportionately high incidence of TBI due to high-risk-taking activities, whereas older adults are particularly susceptible due to age-related vulnerability and increased likelihood of falls [6].

At the biomechanical level, TBI results from the interplay between external forces and individual brain tissue properties. When the head undergoes normal movements such as rotation, the brain is protected from potential damage by the cushioning effect of the meninges and the cerebrospinal fluid (CSF) [7]. The brain exhibits viscoelastic properties, suggesting that its deformation under stress conditions is both time-dependent and non-linear [8]. Under high strain rates, during high-energy impacts or blast exposures, brain tissue demonstrates marked stiffness and mechanically distinct behavior from that observed under gradual loading [9,10]. The types of forces developed during head trauma, namely shear, tensile, and compressive, produce heterogeneous patterns of brain tissue damage that affect cortical and subcortical junctions, the corpus callosum, and deep white matter pathways, and rely on impact direction, magnitude, and duration [11].

Among the manifestations of these biomechanical forces, diffuse axonal injury (DAI) is considered one of the most critical. Unlike focal lesions or hematomas, DAI is a hallmark of rotational acceleration and deceleration injuries, where the shear and torsional forces that develop disrupt the axonal integrity, especially in deeper structures such as the corpus callosum, brainstem, and white matter tracts [12]. At the microscopic level, axonal injury is initiated by mechano-transduction pathways, including microtubule disruption, axolemmal damage, and axonal transport failure, which leads to secondary swelling and disconnection [13]. These pathological cellular processes are accompanied by accumulated proteins such as amyloid precursor protein and tau, which may lead to neurodegenerative diseases [14].

### 1.2. TBI Classification (Severity, Type, and Temporal Phases)

There are several ways to classify a TBI, based on injury severity, neuroimaging, pathophysiology, prognostic models, or the duration of retrograde amnesia [15]. One of the most common methods for determining injury severity is the Glasgow Coma Scale (GCS), first introduced in 1974, used as a clinical tool to evaluate a patient’s level of consciousness through eye, motor, and verbal responses [2,16]. Based on GCS, traumas are categorized as mild (scores 13–15), moderate (scores 9–12), or severe (scores 3–8). However, this classification system has several limitations, since it does not account for the underlying pathology, as patients with divergent imaging findings may present with smaller scores. To address this issue, an updated framework definition was proposed by Manley et al., 2025 [17]. This recent revision promotes a four-pillar system, including clinical examination (GCS with pupillary reactivity), biomarkers (blood-based assays), imaging (structural and functional measures), and modifiers (patient-specific factors that influence outcomes). The Clinical, Biomarker, Imaging Modifiers (CBI-M) framework provides a more holistic approach to TBI classification.

TBI can also be classified based on injury type, which is divided into focal and diffuse lesions. Focal injuries are typically characterized by localized structural damage such as contusions, hematomas, and penetrating lesions, most often confined to cortical regions [18]. In contrast, diffuse injuries present with widespread disruption of axonal networks and microvascular structures across multiple brain areas [19]. Diffuse axonal injury (DAI) is a characteristic pathology resulting from the shear and tensile forces generated during rapid acceleration and deceleration or rotational head movements [20]. In clinical presentation, both focal and diffuse widespread injury mechanisms often co-exist.

Another significant classification is based on the temporal evolution. The primary injury occurs at the moment of impact and reflects the immediate mechanical disruption of neurons, glia, and vasculature [21]. As a result, our research focuses on the secondary injury cascade, which starts from minutes to years post-trauma. While primary injury can be mitigated through preventative measures, such as helmets and seatbelts, it is not amenable to direct medical intervention [22]. Acute secondary injury events unfold with blood–brain barrier (BBB) breakdown, edema, hemorrhage, excitotoxicity, and hypoperfusion [23]. Over subsequent days to weeks, progressive mechanisms such as oxidative stress, mitochondrial dysfunction, axonal degeneration, and neuroinflammation lead to cellular loss [24]. In the chronic phase, these mechanisms converge on persistent neurodegeneration characterized by abnormal protein aggregation, synaptic dysfunction, and long-term cognitive and behavioral decline, features that overlap with neuropathological hallmarks of Alzheimer’s disease (AD) and chronic traumatic encephalopathy (CTE) [25]. Beyond neuropathology, TBI represents the most prevalent neurological disorder worldwide and is associated with a broad spectrum of persistent symptoms. Survivors experience enduring cognitive deficits, emotional and behavioral disturbances, and somatomotor impairments that persist for years after the initial affliction [26]. Sleep disturbances are more common in patients experiencing insomnia, hypersomnia, and poor sleep quality than in the general population [27]. Understanding these temporal and pathological dimensions of TBI is crucial for mapping its overlap with chronic neurodegenerative diseases such as AD.

### 1.3. Alzheimer’s Disease (AD): Overview and Pathology

Alzheimer’s disease (AD) is the most common form of dementia, accounting for 60–80% of all dementia cases worldwide [28,29]. Clinically, AD is characterized by progressive cognitive decline, memory impairment, and behavioral disturbances that compromise daily functioning significantly [30]. From a neuropathological perspective, AD is defined by the accumulation of extracellular amyloid-beta (Aβ) plaques and intracellular neurofibrillary tangles (NFTs) composed of hyperphosphorylated tau protein [31]. These pathological features contribute to synaptic dysfunction, neuronal death, and cortical atrophy in the hippocampus [32]. Although Aβ and tau are considered the central pathological features of AD, its pathophysiology is more complex. Evidence implicates additional molecular mechanisms, including brain insulin resistance, chronic oxidative stress, mitochondrial dysfunction, and persistent neuroinflammation [33]. Dysregulated insulin signaling impairs neuronal glucose utilization, while oxidative stress and mitochondrial damage compromise cellular energy metabolism, leading to synaptic failure [34]. Neuroinflammation processes, mediated by activated microglia and astrocytes, contribute to neuronal injury through the release of pro-inflammatory cytokines, chemokines, and reactive oxygen species (ROS) [35]. Furthermore, vascular abnormalities have a critical role in driving neurodegenerative progression. Disrupted cerebral blood flow compromises oxygen and glucose delivery to neurons, while BBB breakdown allows the infiltration of peripheral immune cells and circulating inflammatory mediators into the central nervous system (CNS). These neurovascular processes increase neuroinflammation, impair the clearance of neurotoxic proteins, and promote neuronal dysfunction, gradually leading to cognitive decline.

### 1.4. Epidemiological Link Between TBI and Neurodegenerative Diseases

Epidemiological studies have demonstrated a strong connection between TBI and the subsequent development of neurodegenerative diseases such as Alzheimer’s disease [36]. A large-scale, propensity-matched cohort study of over 170,000 U.S. military veterans demonstrated that dementia risk increased proportionally with TBI severity, with adjusted hazard ratios of 2.36 for mild TBI without loss of consciousness, 2.51 for mild TBI with loss of consciousness, and 3.77 for moderate-to-severe TBI [37].

A meta-analysis further confirmed that TBI almost doubles the risk of all-cause dementia (OR = 1.79, 95% Cl: 1.66–1.92), while also increasing the risk of AD (OR = 1.60, Cl: 1.44–1.77), vascular dementia (VaD OR = 2.03, 95% CI: 1.79–2.30), and frontotemporal lobar degeneration (FTLD OR = 3.99, 95% CI: 2.20–7.20) [38]. These findings highlight that TBI could be considered a modifiable risk factor for late-life cognitive decline. Human neuropathological studies suggest that TBI can directly initiate or accelerate AD-like processes, including neuroinflammation, excitotoxity, cerebrovascular dysfunction, and BBB breakdown [39]. As stated in the literature, TBI exacerbates the upregulation of amyloid precursor protein (APP) and tau hyperphosphorylation, which are core characteristics of AD [40]. These epidemiological and neuropathological findings support the hypothesis that TBI may serve not only as a trigger but also as a potential accelerator of Alzheimer’s disease pathology.

### 1.5. Study Aim

This review endeavors to provide a comprehensive mechanistic and clinical perspective of the association between traumatic brain injury (TBI) and subsequent neurodegeneration. Increasing evidence suggests a causal link between TBI and the initiation or acceleration of Alzheimer’s disease. The synthesis of epidemiological, neuropathological, and molecular evidence aims to show how acute and repetitive head injuries lead to long-term cognitive decline and the development of chronic neurodegenerative diseases. A crucial objective of the review is to highlight the role of biomarkers in facilitating early detection of TBI-related neurodegenerative risk, while also presenting emerging therapeutic solutions that may mitigate or prevent the progression from acute injury to chronic disease.

## 2. Traumatic Brain Injury vs. Chronic Traumatic Encephalopathy vs. Alzheimer’s Disease

### 2.1. Physical and Neuropathological Consequences of TBI

Traumatic brain injury represents a highly heterogeneous condition characterized by diverse patterns of neuropathological events, with its severity and long-term outcomes determined by the initial biomechanical impact, the magnitude of secondary injury mechanisms, and the brain’s intrinsic capacity for tissue repair. Immediately after trauma, both diffuse and focal structural damage, often accompanied by vascular disruption, predominate. Over time, these acute processes evolve and can give rise to progressive neurodegenerative cascades that unfold over years to decades. As a result, the neuropathological sequelae of TBI are best understood by considering both the injury severity and the temporal trajectory from the acute to chronic phase.

TBI is increasingly recognized as a trigger for progressive neurodegeneration and increases susceptibility to Alzheimer’s disease (AD), Parkinson’s disease (PD), motor neuron disease (MND), and chronic traumatic encephalopathy (CTE) [41,42]. Diffuse axonal injury (DAI) emerges as a common pathological feature of TBI, particularly affecting white matter due to mechanical loading and shear forces generated during impact [14]. DAI pathology begins with primary cytoskeletal disruption, impaired axonal transport, and axonal swelling, which progress to secondary axonal disconnection [43]. These processes can ultimately lead to Wallerian degeneration and proteinopathies involving amyloid-beta and tau [44]. In humans, establishing direct causal evidence linking DAI to downstream neurodegeneration remains challenging, as conventional imaging techniques cannot monitor axonal injury at the cellular level [45]. However, diffusion magnetic resonance imaging (MRI) facilitates in vivo quantification of axonal injury and longitudinal atrophy [46].

DAI Pathophysiology

Mechanical indicators such as von Mises stress, which describes brain tissue distortion, are also used to model the likelihood of DAI in TBI [11]. Primary mechanical injury following trauma involves the rapid deformation of white matter tracts, resulting in cytoskeletal failure and the formation of axonal varicosities and bulbs, i.e., “retraction balls,” which show disconnection. Stretching these axonal fibers to their limit at a rate of 10 s^−1^ can lead to mechanical damage and secondary injury, resulting in neuronal cell death [11]. This secondary phase involves calcium influx and calpain activation, leading to spectrin and microtubule degradation, mitochondrial swelling, and progressive cytoskeletal breakdown over several hours [47]. Experimental injury models suggest that DAI may serve as a central initiating factor for neurodegeneration after TBI. Disrupted axonal transport also leads to intra-axonal accumulation of amyloid precursor protein (APP), along with β-secretase (BACE1) and presenilin-1, promoting intra-axonal amyloid-beta generation [48,49]. Tau further dissociates from microtubules, undergoes hyperphosphorylation, and propagates into a prion-like manner across the neuronal networks [50].

From Axonal Injury to Neurodegeneration

DAI is observed across the full spectrum of TBI severity, from mild concussion to severe injuries, and manifests clinically as transient loss of consciousness or confusion to persistent coma and cognitive dysfunction [14]. Histopathological evidence shows that APP accumulation in damaged axons can be detected within hours upon TBI and can often be more sensitive than silver staining, identifying DAI [51,52]. However, axonal degeneration and white matter loss persist for weeks to years following injury in certain patients [53]. As a result, two non-exclusive mechanistic pathways are proposed to underlie chronic degeneration following TBI: (i) Wallerian degeneration of axons characterized by transport failure; (ii) proteinopathies, particularly amyloid-beta and tau, originating from traumatic axonal pathology and propagating across interconnected brain networks.

Neuroimaging Evidence Linking DAI to Neurodegeneration

Graham et al. 2023 [54] investigated whether the severity and spatial distribution of diffuse axonal injury (DAI) predict progressive neurodegeneration in a cohort of 55 patients with moderate-to-severe TBI. Using diffusion MRI, the study demonstrated that the DAI burden is the leading factor of chronic white degeneration, promoting a causal relationship between axonal injury and subsequent tissue loss. Fractional anisotropy (FA), a diffusion-derived metric used to calculate the white matter structure, has emerged as an important marker of axonal integrity in the chronic phase of TBI [55]. Reduced FA reflects axonal disruption and associated neuroinflammation and has been correlated with large-scale network dysfunction and cognitive impairment [56]. Serial T1-weighted volumetric imaging further enables accurate quantification [57]. Diffusion abnormalities have also been linked to gray matter volume changes through network diffusion modeling, indicating the relationship between white matter disconnection and cortical degeneration [58]. Conventional imaging markers of DAI, including T2, fluid-attenuated inversion recovery (FLAIR), and T2*-weighted sequences, have been associated with region-specific atrophy pathways early after injury [45].

Overall, DAI has emerged as a strong predictor of progressive neurodegeneration after moderate-to-severe TBI, explaining most of the variability in white matter atrophy and long-term neurodegeneration. Evidence from both pathology and neuroimaging supports the central role of axonal injury in progressive tissue loss, functional impairment, and finally increased dementia risk.

#### 2.1.1. TBI Types and Severity

The clinical spectrum of TBI is classified as mild, moderate, or severe based on the Glasgow Coma Scale (GCS), the duration of loss of consciousness (LOC), and the extent of post-traumatic amnesia [59]. Mild TBI is often described as a concussion and typically presents with transient LOC, confusion, and subtle cognitive changes. While symptoms usually resolve, a subset of patients develop persistent post-concussive complaints [60]. Moderate-to-severe injuries are characterized by extended loss of consciousness, radiologically visible lesions such as contusions or hematomas, and a greater risk of long-term disability [61,62]. At the pathological level, severe TBI is associated with diffuse axonal injury (DAI), hemorrhage, and necrotic cavitation [12]. Even in mild cases, microscopic axonal disruption and glial reactivity may occur, changes that are not readily detectable on conventional imaging but have functional consequences [63].

#### 2.1.2. Acute Phase of TBI

The acute phase of TBI progresses within minutes to days after the initial affliction and involves a cascade of cellular, vascular, and metabolic disturbances [64]. Mechanical deformation and shearing forces disrupt neuronal and glial membranes, causing further ionic disequilibrium, glutamate release, and excitotoxic activation of N-methyl-D-aspartate (NMDA) and α-amino-3-hydroxy-5-methyl-4-isoxazolepropionic acid (AMPA) receptors [65]. This cascade leads to calcium overload, mitochondrial dysfunction, and excessive production of reactive oxygen and nitrogen species, driving oxidative stress and energy depletion. Cytotoxic edema develops as astrocytic and neuronal swelling accumulate, exacerbating intracranial pressure and perfusion deficits [66]. At the same time, the blood–brain barrier becomes compromised and permeable as tight-junction proteins degrade, endothelial adhesion molecules are upregulated, and peripheral immune cells infiltrate the parenchyma, amplifying neuroinflammatory responses [67,68]. Microvascular dysfunction, microthrombosis, and early hypoperfusion enhance the secondary injury cascade, indicating chronic pathology.

#### 2.1.3. Chronic Phase of TBI

Beyond the acute phase of TBI, many survivors undergo a chronic phase characterized by long-lasting neuropathological changes that all contribute to progressive cognitive, behavioral, and motor deficits. Diffuse axonal injury evolves into chronic axonopathy associated with disconnected white matter tracts, demyelination, and gliosis. Persistent microglial activation and astrocytosis maintain a pro-inflammatory environment for years following even a single moderate-to-severe TBI, as shown by both human postmortem studies and in vivo imaging [69]. These inflammatory processes converge with vascular injury, blood–brain barrier disruption, and problematic clearance pathways to drive abnormal protein accumulation, particularly hyperphosphorylated tau and amyloid-beta deposits [19]. This results in a neuropathological process that links TBI with later neurodegenerative diseases, including chronic traumatic encephalopathy and Alzheimer’s disease. Clinically, the chronic phase presents with enduring cognitive dysfunction, psychiatric disturbances, namely depression and anxiety, and increased risk of dementia.

#### 2.1.4. Diffuse Axonal Injury (DAI)

Diffuse axonal injury (DAI) is recognized as one of the most characteristic substrates of TBI, particularly in acceleration-deceleration and rotational injuries, and primarily affects the axons of cerebral white matter, which are vulnerable to mechanical strain during trauma [43]. Under a pathological spectrum, DAI represents not a single lesion but a spectrum of axonal damage ranging from cytoskeletal disruption and impaired axonal transport to swelling and axonal disconnection, further leading to Wallerian degeneration. The hallmark histological findings include axonal swellings and bulbs, with amyloid precursor protein (APP) accumulating within hours of injury. Although the definitive diagnosis still requires post-mortem histopathology, innovative neuroimaging methods such as diffusion tensor imaging (DTI) have been presented as sensitive tools used in vivo to detect white matter abnormalities, with fractional anisotropy (FA) reduction being a sensitive marker of axonal injury [70].

Experimental evidence suggests that DAI is a trigger of progressive neurodegeneration and not only an acute injury phenomenon. Cytoskeletal disruption initiates axonal swelling and bulb formation in sites where APP accumulates with β-secretase (BACE1) and presenilin-1, producing amyloid-β [71]. Dissociation from microtubules and abnormal phosphorylation following injury were also seen in young athletes and experimental mouse models, showing that traumatic axonal injury can induce early tauopathy [72]. Recent studies showed prion-like propagation of tau pathology in animal models of TBI, suggesting a potential mechanistic link between DAI and progressive neurodegeneration. A single moderate-to-severe TBI could accelerate dementia onset and increase the risk of AD through the same axonal pathways of protein accumulation and degeneration. Imaging studies show that DAI predicts progressive brain atrophy after TBI. Using diffusion MRI and volumetric T1 imaging, Graham and colleagues demonstrated that fractional anisotropy in the chronic phase following TBI was a strong predictor of subsequent atrophy in white matter tracts such as the corpus callosum [44]. In addition, calcium influx and calpain activation following axonal stretch injury trigger proteolysis of structural proteins, disruption of microtubules, and mitochondrial dysfunction, which all lead to axonal failure [73]. These inflammatory processes may persist for years following trauma in the corpus callosum [53]. DAI presents as a critical mechanical and imaging marker of progressive post-traumatic neurodegeneration, since it establishes a link between axonal disruption and chronic degeneration.

#### 2.1.5. Chronic Traumatic Encephalopathy (Neuropathological Features, Clinical Manifestation, Risk Factors, and Diagnosis)

Chronic traumatic encephalopathy (CTE) is a progressive neurodegenerative tauopathy associated with repetitive head impacts (RHIs) [74]. Originally, it was described in boxers as ‘punch drunk’ syndrome; this condition is now well-recognized across a range of contact sports and military personnel [75]. It is neuropathologically unique as it consists of perivascular deposits of hyperphosphorylated tau found at the depths of cortical sulci [76]. Clinically, CTE manifests with cognitive, mood, and behavioral alterations that can manifest years after exposure. The first descriptions of long-term neurological impairment following repeated head trauma were reported by Martland in 1928, who described boxers’ symptomatology with gait disturbance, slowed speech, and cognitive decline [77]. Millspaugh later described the punk drunk syndrome as dementia pugilistica [78]. In 1973, Corsellis and colleagues published a neuropathological study of 15 retired boxers identified as having cortical atrophy, neurofibrillary tangles, and cerebellar scarring, further enhancing the theory of dementia pugilistica [79]. These early observations set the basis for later recognition of a neurodegenerative disease. In the 2000s, the work of McKee and Colleagues established modern diagnostic criteria and a four-stage neuropathological staging scheme [80,81]. Early disease presents with focal perivascular lesions, while advanced stages show widespread cortical, limbic, and brainstem involvement [82].

The central lesion in CTE is the perivascular deposition of hyperphosphorylated tau (p-tau) in neurons and astrocytes, located deep in the cortical sulci [83]. This is the most important difference compared to AD, because although both are tauopathies, CTE lesions are perivascular, sulcal, and not associated with neuritic amyloid plaques. Molecular studies, using cryo-electron microscopy, showed that tau filaments in CTE adopt a distinct conformation compared to those in AD, proposing CTE as a unique tauopathy [84]. Although tau remains the defining pathology, other findings include transactive response DNA-binding protein 43 (TDP-43) inclusions, axonal degeneration, and white matter degradation [85]. These changes overlap with other neurodegenerative diseases, further highlighting the need for specificity in biomarker CTE-tailored development. Systematic comparisons between CTE and normal aging, as well as other tauopathies, validate the specificity of perivascular sulcal tau lesions [86]. Specifically, the presence of TDP-43 and amyloid-related pathology in both CTE and non-CTE brains further highlights the importance of distinct pathological interpretation. A recent study, which examined 185 athletes, demonstrated cortical thinning, neuronal loss, and synaptic alterations, particularly in sulcal regions, further enhancing the hypothesis that regional biomechanical vulnerability determines lesion localization [87]. Increased astrocytic activity and aquaporin-4 upregulation around the lesions showed BBB dysfunction; however, microgliosis was less prominent [88].

Clinical Manifestations

The clinical presentation of CTE is varied. Symptoms present years after injury and cluster into four main domains: (i) cognitive with executive dysfunction and memory loss, (ii) behavioral with impulsivity, (iii) mood with psychiatric disorders such as depression and suicidality, (iv) motor with Parkinsonian characteristics and dysarthria [89,90,91]. Clinical studies of former football players showed a high prevalence of behavioral and mood changes before cognitive decline [92,93]. To explain the gap between neuropathology and clinical presentation, Montenigro et al. 2014 [94] proposed the concept of traumatic encephalopathy syndrome (TES). 

Risk Factors

The principal risk factor for CTE is exposure to repetitive head trauma. Mez et al. 2017 [95] reported CTE pathology in 87% of 202 deceased former American football players, with disease severity being directly correlated to their athletic career duration. Alosco et al. 2018 [96] further demonstrated a dose–response relationship between years of football and risk of CTE, which was thought to be independent from concussion history. Other modifiers include genetic risk factors such as APOE and TMEM106B, although these associations are still under investigation [97,98].

Diagnosis

At present, CTE can only be diagnosed definitively at autopsy. The DIAGNOSE CTE Research project, a prospective study of 240 men with different exposures to American football, aims to develop clinical criteria and biomarkers [99]. Fluid biomarkers, namely plasma tau, neurofilament light chain, and markers of microglial activation (soluble triggering receptor expressed on myeloid cells 2 [sTREM 2]), are also being evaluated [100].

### 2.2. Molecular and Cellular Mechanisms Linking TBI to AD

#### 2.2.1. Blood–Brain Barrier Dysfunction

The blood–brain barrier (BBB) is a specialized neurovascular unit formed by endothelial cells, pericytes, and astroglial cells, which together maintain central nervous system homeostasis [101]. Following TBI, the BBB undergoes rapid and sustained disruption, which exacerbates secondary injury cascades. Mechanical shear stress and vascular rupture comprise barrier integrity, allowing plasma proteins and immune cells to enter the parenchyma [39]. Endothelial cell activation upregulates adhesion molecules such as intercellular adhesion molecule (ICAM), vascular cell adhesion molecule-1 (VCAM-1), and selectins, facilitating leukocyte adhesion and transmigration, which further amplifies neuroinflammation and oxidative stress cascade [102]. Elevated P-selectin levels promote multicellular aggregation and microvascular occlusion, aggravating ischemic vulnerability. Vascular endothelial growth factor (VEGF) rises sharply after TBI, inducing angiogenesis but simultaneously downregulating tight junction proteins such as occludin and claudin-5, weakening endothelial cohesions and increasing vascular leakage [103,104]. These alterations result in vasogenic edema, neuroinflammation, and impaired tissue repair capacity. BBB disruption is not transient, since chronic dysfunction is characterized by pericyte loss, endothelial degeneration, and tight junction protein disorganization [105]. Chronic mural cell degeneration and reduced expression of Caveolin-1 (Cav-1) and low-density lipoprotein receptor-related protein 1 (LRP1) impair Aβ and tau clearance, which establishes neurodegeneration [106].

Findings from both human and animal studies suggest that the BBB disruption is a central link between TBI and Alzheimer’s disease. Evidence indicates BBB breakdown in AD precedes cognitive decline, manifesting as vascular leakage, reduced glucose transport, and leukocyte infiltration [107]. In TBI, the “two-hit” hypothesis model proposes that the initial mechanical disruption is compounded by secondary inflammatory and metabolic cascades, altering vascular and cellular permeability [108,109]. Pharmacological interventions targeting BBB repair, even one year post-injury, reversed axonal degeneration and restored cognition, highlighting barrier restoration as a promising therapeutic strategy [110].

At the molecular level, BBB vulnerability is shaped by genetic and signaling factors. In APOε4 carriers, increased BBB permeability has been linked to cyclophilin A/matrix metalloproteinase 9 (CypA/MMP-9) pathway activation and LRP1 degradation. Concurrently, reduced expression of the major facilitator superfamily domain-containing protein 2A (Mfsd2a) promotes caveolae-mediated transcytosis, further compromising barrier integrity [111]. Impairment of Wingless/Integrated (Wnt)/β-catenin signaling further impairs tight junction integrity and endothelial-pericyte communication, linking genetic and molecular predisposition to blood–brain barrier fragility [112].

#### 2.2.2. Chronic Vascular Changes

Beyond the parenchymal injury, TBI triggers early alterations in leptomeningeal arterial vascular smooth muscle cells (VSMCs) that disrupt cerebrovascular homeostasis and promote amyloidogenic processes. Experimental and post-mortem studies have demonstrated pathological changes in cerebral vessels, including altered distribution of NOTCH3 and alpha-smooth muscle actin (α-SMA) within the tunica media, indicating vascular smooth muscle cell dysfunction. These structural abnormalities are accompanied by the early aggregation of Aβ peptides (Aβ1-40/42, Aβ1-16, beta C-terminal fragment of APP (β-CTF)/C99) around VSMCs, even in young individuals following TBI, whereas controls show strong uniform marker expression and minimal vascular amyloid deposition [113]. These vascular changes are thought to arise from local hypoxia and oxidative stress, which lead to beta-secretase-mediated (BACE-1) APP cleavage with relative downregulation of the non-amyloidogenic a disintegrin and metalloproteinase domain-containing protein 10 (ADAM10) pathway. Therefore, VSMC dysfunction impairs cerebrovascular reactivity, compromises cerebral perfusion, and might lead to secondary ischemia and long-term neurodegeneration [114].

#### 2.2.3. Excitotoxicity

Mechanical forces generated upon head impact strain the neuronal and glial membranes, leading to widespread depolarization of axonal networks and excessive glutamate release. Elevated extracellular glutamate will then activate synaptic and extra-synaptic NMDA receptors [115,116]. The inability of the astrocytic transporter to sustain the extracellular glutamate drives Ca^2+^ influx and downstream apoptotic and necrotic signaling [117]. The rapid entry of cations depolarizes neurons, which leads to the indiscriminate release of neurotransmitters such as glutamate and disrupts synaptic integrity and network stability, further contributing to both acute neuronal injury and long-term neurodegenerative vulnerability.

#### 2.2.4. Oxidative Stress

TBI promotes the generation of reactive oxygen species (ROS) and reactive nitrogen species (RNS) through mitochondrial dysfunction, altered cellular metabolism, and inflammatory oxidase activation [118]. The resulting oxidative and nitrosative stress induces lipid peroxidation, protein nitration, and DNA damage. These alterations contribute to neuronal loss, glial reactivity, and neuroinflammation, accelerating neurodegenerative processes [119].

#### 2.2.5. Mitochondrial Dysfunction and Ferroptosis

Mitochondria regulate cellular energy metabolism, redox homeostasis, and Ca2+ regulation, all of which are disrupted following TBI [120]. Impaired electron transport and oxidative phosphorylation (OXPHOS) lead to adenosine triphosphate (ATP) depletion, ROS overproduction, and maladaptive mitochondrial dynamics that compromise repair and synaptic plasticity, which are phenomena observed in early stages of Alzheimer’s disease. A recent study implicated the role of ferroptosis, which is an iron-dependent lipid peroxidation pathway, in TBI-associated neuronal death [121]. In experimental models, pharmacological or genetic inhibition of mitoNEET (CDGSH iron–sulfur domain-containing protein 1 [CISD1]) reduces ferroptosis and preserves cognition by stabilizing dihydroorotate dehydrogenase (DHODH)-mediated mitochondrial defenses, indicating that the mitoNEET/DHODH axis could act as a potential therapeutic target [122]. Region-specific proteomic signatures further reveal that repetitive as opposed to single, mild TBI persistently dysregulates mitochondrial and synaptic proteins, especially within the hippocampus, correlating with cognitive decline [123].

#### 2.2.6. Coagulopathy and Microvascular Hemorrhage

TBI initiates an early hypercoagulable state through BBB disruption, extrinsic pathway activation, endothelial glycocalyx degradation, and platelet leukocyte endothelium interaction, followed by coagulopathy and hyperfibrinolysis [124,125,126,127].

#### 2.2.7. Cerebral Blood Flow Changes

Altered cerebral blood flow is common following TBI and strongly influence prognosis. Hypoperfusion correlates with cognitive impairment and poor outcomes, whereas timely cerebral blood flow (CBF) restoration is associated with functional improvement [128,129]. Loss of vascular muscle cell contractility and impaired pericyte-mediated regulation disrupt neurovascular coupling, since they limit oxygen and glucose delivery in periods of increased metabolic demand [130,131,132].

#### 2.2.8. Genetic Susceptibility Linking TBI and Alzheimer’s Disease

Inherited genetic factors and dysregulated molecular pathways are recognized as important contributors in determining how traumatic brain injury (TBI) and diffuse axonal injury (DAI) progress toward Alzheimer’s disease (AD) and chronic traumatic encephalopathy (CTE) [133]. Recent studies have expanded the understanding of how somatic mutations, trauma-related alterations in lipid metabolism, protein clearance pathways, and blood–brain barrier (BBB) regulators may accelerate neurodegeneration after head injury [134]. Somatic mosaicism has recently been identified as a potential mechanism linking acute trauma to chronic neurodegeneration. Single-cell sequencing of TBI and AD brain tissue revealed overlap in somatic mutations across molecular pathways, which suggests that trauma may accelerate genomic instability in neurons and glia. This analysis highlighted that trauma is not only a mechanical insult but also a mutagenic trigger that may exacerbate age-related genetic vulnerabilities [135].

Among germline susceptibility factors, the APOE locus remains the most extensively studied. The ε4 allele was associated with worse outcomes following TBI and an increased risk of AD [136]. Previous studies have shown a tenfold increased risk of AD diagnosis among ε4 carriers with a TBI history [137]. A meta-analysis of 2600 patients suggested that ε4 carriers have poorer global outcomes following TBI [138]. The structural and vascular consequences of APOE isoforms show that ε4 exacerbates cerebral amyloid angiopathy, BBB leakage, and perivascular tau deposition. The human apolipoprotein E gene, APOE, encodes three isoforms—APOEε2, APOEε3, and APOEε4—and is predominantly expressed by astrocytes in the brain. APOEε4 has been linked with being the strongest genetic risk factor for early- and later-onset AD, as well as poor outcomes following TBI, since it disrupts the BBB [139]. APOE ε4 interacts with lipid metabolism and neuroinflammatory cascades, accelerating both the traumatic and degenerative pathology, whereas ε2 appears protective in certain cases but is a risk factor in severe vascular injury. The age of AD onset is three years lower among ε4 carriers with TBI compared to non-carriers with TBI [140]. APOE’s role in oxidative stress and excitotoxic process after trauma enhances the hypothesis that isoform-specific effects extend to acute injury physiology and long-term degeneration.

New molecular mechanisms within the APOE locus have also been identified. The translocase of outer mitochondrial membrane 40 (TOMM40)-APOE (T9A2), a chimeric fusion transcript, reportedly modified mitochondrial trafficking and accelerated amyloidogenic processing in AD models [141]. Given the importance of mitochondrial dysfunction in TBI pathology, the TOMM40-APOE fusion may represent an important molecular link between acute axonal injury and late amyloid-driven degeneration.

Autophagy and protein clearance pathways also have a central role in linking TBI to subsequent neurodegenerative pathology. BCL2-associated athanogene 3 (BAG3), a co-chaperone that regulates autophagy–lysosome flux, plays a key role in tau clearance. Reduced BAG3 function disrupts tau regulation and accelerates neurofibrillary pathology, which enhances the hypothesis that repetitive TBI, through impaired proteostatic recovery, may increase vulnerability to CTE and AD tauopathy [142]. Another study showed that genetic variants affecting neprilysin activity have been shown to reduce enzymatic clearance of Aβ, thereby potentiating plaque accumulation. These findings suggest that genetic regulation of proteostasis is important in determining whether traumatic brain injury progresses towards AD pathology [143,144]. Additional susceptibility loci have also been identified through genome-wide association studies (GWASs), including variants in Triggering Receptor Expressed on Myeloid Cells 2 (TREM2). In particular, the R47H variant of TREM2 has been associated with a fivefold increased risk of Alzheimer’s disease (OR = 5.05, 95% CI: 2.77–9.16, *p* < 0.001) [145].

Signaling pathways that regulate BBB integrity and neurovascular stability present as essential regulators of TBI to AD progression. Transforming growth factor beta (TGF-β) signaling, which normally has a neuroprotective role since it promotes the expression of tight junction proteins and mediates the recruitment of astrocytes to the BBB, can become maladaptive under chronic trauma conditions, while promoting glial scar formation and impaired repair [146]. Mfsd2a, a lipid transporter crucial for BBB integrity, is downregulated following a TBI, potentially facilitating amyloid influx and vascular inflammation [147]. In particular, this transporter downregulates with age, with a stronger reduction in 5xFAD Alzheimer’s disease (AD) model mice compared to wild-type mice. This downregulation is amplified further in the presence of APOE4, a major AD risk allele, and a similar reduction is also observed in human APOE4, suggesting that enhancing Mfsd2a expression could potentially restore BBB integrity [148]. The Wnt/β-catenin signaling pathway, which regulates endothelial tight junctions and cellular proliferation, is disrupted in both AD and TBI, contributing to vascular dysfunction. As a result, the upregulation of Wnt/β-catenin signaling is important to vascular repair, as shown with the intranasal application of recombinant Wnt3a, which promoted BBB repair after TBI [149]. Matrix metalloproteinase-9 (MMP-9) is upregulated following trauma and contributes to BBB disruption and neuronal apoptosis. In TBI, MMP-9 expression is significantly elevated in neutrophils after the initial insult [150]. Sonic the Hedgehog (Shh) signaling is pivotal in neural development and normal brain function. However, in TBI, it has been implicated that endogenous Shh expression is downregulated after TBI, leading to impaired signaling in the acute phase and suppressed neural stem cell renewal in chronic TBI [151]. Finally, mechanistic (mammalian) targeting of rapamycin (mTOR) dysregulation has been observed in TBI, with hyperactivation linked to impaired autophagy and accumulated toxic protein aggregates. Evidence indicates that mTOR activation occurs early in Alzheimer’s disease pathogenesis, with upregulated mTOR signaling promoting Aβ and tau aggregation, neuroinflammation, and oxidative stress [106].

### 2.3. Clinical Presentation of TBI-Related Neurodegeneration

The clinical spectrum of traumatic brain injury-related neurodegeneration (TBI-ND) includes a plethora of cognitive, behavioral, and psychiatric manifestations that can impair the quality of life of the individual. Memory impairment is frequently reported in the literature, with patients experiencing difficulties in learning, retaining, and retrieving new information [152,153]. Both anterograde and retrograde amnesia were observed, disrupting the hippocampal cortical network [154]. Executive dysfunction with impaired decision-making processes and organization was also recorded [155]. Beyond cognitive decline, mood disturbances and behavioral dysregulation were common clinical features [156]. Irritability, agitation, depression, and anxiety emerge, which disrupt the interpersonal relationships of the individual [157].

### 2.4. Risk Factors

TBI does not operate as an isolated affliction but instead as a contributor that interacts with multiple biological and demographic risk factors to accelerate the progression towards Alzheimer’s disease and other neurodegenerative diseases [158], including age, sex, vascular and systemic comorbidities, sleep disturbances, and neuroinflammation.

#### 2.4.1. Age

Age is a crucial determinant of dementia risk in the general population and modifies the effects of TBI on long-term outcomes. Epidemiological data suggest that younger individuals experience a disproportionately higher relative risk. A meta-analysis of 32 observational studies found that TBI was associated with a pooled risk ratio (RR) of 1.51 (95% CI: 1.26–1.80) for developing Alzheimer’s disease [159]. Gardner et al. (2014) [160] similarly reported that moderate-to-severe TBI reported nearly double the risk of dementia in older adults, while mild TBI showed weaker associations. Interestingly, age was also found to suppress the association between traumatic brain injury severity and functional outcomes. Adding age at injury to regression models significantly increased the association between TBI severity and functioning at all follow-up points (up to 10 years) [161]. These findings support the hypothesis that TBI accelerates brain aging by lowering the threshold for clinical dementia onset. Recent neuroimaging evidence supports this biological aging model, as mild TBI patients demonstrate significantly increased brain-predicted age gaps (brain-PAGs) correlated with cognitive impairment and neurofilament light chain levels [162].

#### 2.4.2. Sex and Gender

Sex differences in TBI incidence are increasingly being investigated in the literature, reflecting interest in potential demographic influences on injury patterns. Men sustain more TBIs overall, with peaks in young adulthood and late life, but women experience worse functional and cognitive consequences [163]. A meta-analysis of 19 studies showed that female patients demonstrated poorer outcomes in 85% of examined domains, with a small-to moderate effect size (d = −0.15) [164].

Another study found that female athletes with sports-related concussion reported more severe post-concussive symptoms and slower recovery trajectories than males [165]. Although some studies indicate better psychosocial adjustment in women, most of the evidence points towards sex-specific vulnerabilities, reflecting hormonal influences and immune regulation.

#### 2.4.3. Vascular and Systemic Comorbidities

TBI shares common vascular and systemic risk factors with AD, including hypertension, diabetes, and hyperlipidemia [166]. Hypertension exacerbates blood–brain barrier (BBB) breakdown, a hallmark of both TBI and AD, and is associated with worse cognitive outcomes after head trauma [167]. Diabetes mellitus promotes amyloidosis and oxidative stress, while it exacerbates the TBI-induced cortical damage and is associated with increased mortality and poorer recovery [168]. Hyperlipidemia and atherosclerosis promote amyloid deposition and tau hyperphosphorylation, while high-fat diets in experimental TBI models delay BBB repair and worsen cognitive outcome [169].

#### 2.4.4. Lifestyle Exposures

Modifiable lifestyle factors increase the risk of dementia and worsen post-TBI recovery. Smoking is associated with vascular dysfunction, oxidative stress, and increased tau phosphorylation in AD models, while it is linked with poorer neurocognitive outcomes after TBI [170,171]. Alcohol use increases BBB permeability, exacerbates amyloid β-aggregation, and contributes to higher mortality after TBI.

#### 2.4.5. Sleep and Neuroinflammation

Sleep disturbance and neuroinflammation are important mechanisms through which TBI predisposes towards AD. Sleep issues are observed in most AD patients and frequently precede cognitive decline [172]. In TBI, sleep disturbances are highly prevalent and linked with increased neuroinflammation [173]. Green et al.’s 2020 [174] study highlighted, among others, how cytokines such as interleukins, IL-1β and IL-6, and TNF-α, which normally act as sleep regulators, upregulate after trauma, increase microglial activation, and ultimately exacerbate secondary brain injury. 

#### 2.4.6. Neurovascular Dysfunction

The neurovascular unit (NVU) provides a convergent mechanism linking these diverse risk factors. Genetic susceptibility, vascular changes, systemic exposures, and TBI resulting in mechanical damage are linked with BBB integrity disruption and cerebral flow regulation. This dysfunction allows the entry of plasma proteins, namely fibrinogen, into the CNS, triggers chronic neuroinflammation, and accumulates amyloid and tau [175]. Hippocampal CA1 pyramidal neurons exhibit selective neuronal vulnerability in the setting of NVU dysfunction. TBI-related disruption of NVU integrity explains why survivors show increased susceptibility to hippocampal-dependent memory decline characteristic of AD.

### 2.5. Biomarker Evidence

#### 2.5.1. Fluid Biomarkers

Fluid-based biomarkers in cerebrospinal fluid (CSF) and blood are critical in traumatic brain injury pathophysiology. Among the most investigated are amyloid-β (Aβ), total tau (t-tau), phosphorylated tau (p-tau), neurofilament light chain (NfL), glial fibrillary acidic protein (GFAP), ubiquitin carboxy-terminal hydrolase L1 (UCH-L1), and S100B.

These markers reflect axonal degeneration, astroglial damage, amyloid metabolism, and cytoskeletal disruption [176,177]. Acute TBI triggers a cascade of biomarker alterations with distinct temporal patterns. GFAP becomes detectable within the first hour after injury and peaks between 20 and 24 h, and declines over the following 72 h [178]. S100B concentrations are detectable in the peripheral circulation as early as 15 min and return to baseline within 1–2 h [179]. UCH-L1 is detectable in peripheral blood shortly post-injury and increases, reaching a peak 8–12 h after initial trauma [180]. Findings from the large CENTER-TBI cohort of 2869 patients showed that serum levels of GFAP, NfL, NSE, S100B, t-tau, and UCH-L1 are strongly correlated with injury severity and lesion extension with diffuse axonal injury (Marshall III/IV), producing higher concentrations than focal mass lesions [181]. In another study, serum NfL outperformed other biomarkers, namely tau, S100B, and neuron-specific enolase (NSE), in characterizing sports-related concussion (SRC) [182]. Proteomics analyses emphasize the biological aspect of TBI. A study identified 16 plasma proteins altered within 10 days following moderate-to-severe TBI, including neuronal (UCH-L1, visinin protein 1), astroglial (GFAP, S100B), neurodegenerative (tau, pTau231, presenilin-1 (PSEN1), Aβ42), and inflammatory (IL-16, C-C motif chemokine ligand 2 [CCL2]) markers. These molecular alterations correlated with lesion volumes and white matter integrity, underscoring the interplay between biochemical signals and pathology [183]. Recent evidence also demonstrated long-term biomarker changes in athletes exposed to repetitive head impacts. Elevated plasma p-tau217 and NfL were noted in former rugby players compared to controls, with p-tau217 levels associated with traumatic encephalopathy syndrome and smaller hippocampal volume, while NfL was linked to anxiety symptoms [184].

#### 2.5.2. Imaging Biomarkers

Neuroimaging is essential to understand the neuropathological consequences of TBI and RHI, particularly in relation to neurodegenerative diseases such as Alzheimer’s disease and chronic traumatic encephalopathy (CTE). Advances in MRI, positron emission tomography (PET), and fluid biomarker imaging have drastically evolved the ability to detect both acute injuries and long-term neurological changes.

PET imaging facilitates the in vivo detection of hallmark AD pathologies, especially beta-amyloid (Aβ) and tau aggregates. In AD, PET findings showed that plaques emerge in a predictable spatial and temporal sequence [185]. Amyloid PET has been proposed as the earliest biomarker of AD, appearing years before clinical manifestation [186,187]. However, Eagle et al. 2020 [188] reported that middle-aged individuals with a history of repeated TBIs showed no significant elevations in amyloid or tau PET uptake compared with controls, despite a higher symptom burden. These results highlight that conventional AD biomarkers may not reliably differentiate risk groups in trauma-related neurodegeneration; thus, alternative TBI-targeted biomarkers may be needed. MRI techniques provide sensitive markers for traumatic and degenerative processes. Diffusion tensor imaging (DTI) is particularly designed to provide information on axonal injury.

Preclinical models demonstrated DTI’s sensitivity in detecting traumatic white matter damage [189]. Zimmerman et al. [190] identified signs of axonal and vascular injury in elite rugby players, with abnormal trajectories of white matter changes. Volumetric MRI changes reveal reduced whole-brain and hippocampal volume in professional rugby players [191]. Larger studies, such as the BRAIN Health Cohort, are now examining whether volumetric brain loss, white matter changes, and fluid biomarkers (NfL, GFAP, phosphorylated tau) correlate with exposure extent and clinical outcome [192].

Raji et al. 2024 [193] reported that MRI volumetry strongly distinguishes TBI from early- and late-onset Alzheimer’s and behavioral variant frontotemporal dementia (bvFTD) by distinct atrophy patterns. Atrophy is identified in the hippocampus in AD, the frontal lobe in the bvFTD, and white matter loss in TBI. 

Shetty et al. 2015 [194] reviewed the established role of CT and MRI in detecting acute trauma, including cortical contusions, DAI, and secondary complications such as hydrocephalus. While CT remains the first-line modality for acute injuries, MRI provides increased sensitivity for smaller contusions and chronic conditions such as encephalomalacia. Recent advances highlight the important role of imaging in correlation with blood-based biomarkers. Lagramante et al. [195] demonstrated that GFAP and UCH-L1 assays could exclude with accuracy, CT-detectable lesions in suspected mild TBI. Similarly, this is also shown in multimodal studies assessing the relationship between volumetric MRI and white matter integrity as well as plasma biomarkers in retired athletes.

#### 2.5.3. Comparison to Alzheimer’s Disease Biomarker Profiles

An important question in TBI research is whether the biomarker alterations observed following injury resemble those characteristics of Alzheimer’s disease (AD). Both conditions share certain common markers, namely amyloid-β, total tau (t-tau), phosphorylated tau (p-tau), neurofilament light chain (NfL), and glial fibrillary acidic protein (GFAP), but they differ in timing of appearance, magnitude, and long-term pathways [196,197]. In AD, the combined presence of decreased CSF Aβ42, elevated p-tau181 or p-tau217, and increased total tau constitutes a validated biomarker indicator, with detection established in CSF and now increasingly detectable in blood through highly sensitive single-molecular array (Simoa) assays [198,199]. In TBI, acute elevations in GFAP, NfL, and tau reflect immediate structural and axonal injury, while p-tau 217 elevations suggest a convergence with AD pathology [200,201]. However, biomarker pathways appear to differ in magnitude and temporal relationships.

Amyloid pathology emerges early in TBI, where increased APP cleavage and impaired clearance due to lymphatic and blood–brain barrier dysfunction contribute to acute Aβ accumulation [202]. While AD is defined by a consistent reduction in CSF Aβ42 and plasma Aβ42/40 ratio, TBI produces more heterogeneous Aβ dynamics, with acute elevations that may transition into chronic deposition [203]. Recent work by Friberg et al. 2024 [204] demonstrated overlapping CSF profiles between chronic TBI (cTBI) patients and those with AD, indicating a partial shared pathway with amyloid-driven pathology.

Tau-related changes also present an evident overlap between the two conditions. In AD, elevated CSF and plasma p-tau, especially subcategories such as p-tau217 and p-tau231, are highly specific for AD pathology and closely linked to neurofibrillary tangle accumulation [205]. TBI is characterized by acute elevations in total tau (t-tau) reflecting axonal damage, while chronic increases in phosphorylated tau (p-tau217) have been reported in athletes with traumatic encephalopathy syndrome (TES) [206].

Axonal injury markers, particularly NfL, present strong prognostic utility in TBI. Andersson et al. (2024) [207] demonstrated that cerebrospinal fluid (CSF) NfL levels continue to rise for up to 2 weeks following severe TBI and independently predict unfavorable outcomes at both one year and 10–15 years post-injury. Elevated plasma NfL has also been associated with depressive and anxiety symptoms, as well as frontal cortical atrophy, in retired rugby players [205]. Astroglial injury markers, most notably glial fibrillary acidic protein (GFAP), show marked divergence between TBI and AD. In severe TBI, CSF GFAP peaks on days 3–4 and remains elevated for months or years, with higher levels predicting poor long-term outcomes [207]. Synaptic injury markers, including synaptosomal-associated protein 25 (SNAP-25) and visinin-like protein 1 (VILIP-1), are emerging as potential informative biomarkers. Evidence from sTBI indicates a biphasic trajectory, with an early rise reflecting primary synaptic damage and a delayed peak linked to neuroinflammatory responses. Importantly, SNAP-25 and VILIP-1 demonstrated superior predictive performance for six-month outcomes compared to NfL or GFAP [208]. These biomarkers were also elevated in AD and other neurological conditions, highlighting their role as indicators of synaptic dysfunction [209].

Neuroinflammatory markers differentiate TBI from AD. Acute TBI triggers robust increases in cytokines (IL-1β, IL-6, TNF, and IL-17A) and persistent immune activation, with autoantibodies against GFAP, S100B, and Myelin-associated glycoprotein (MAG) detected more than a decade post-injury [210,211]. In AD, inflammation is a prominent feature but tends to follow a slower and more chronic pathway without the acute peaks that are characteristic of TBI.

#### 2.5.4. Neuroinflammation as a Mediator of Tau Pathology

The relationship between traumatic brain injury (TBI) and tau pathology is now being increasingly recognized as a key pathway connecting acute trauma to chronic neurodegeneration. Neuroinflammation driven by microglial activation, cytokine release, and inflammasome signaling is an important mediator of tau phosphorylation and subsequent expansion. This mechanistic link provides biological plausibility to the epidemiological association between TBI and tauopathies such as chronic traumatic encephalopathy and Alzheimer’s disease. Human post-mortem studies demonstrate that even a single moderate-to-severe TBI can lead to widespread phosphorylated tau (p-tau) accumulation, involving cortical layers, the hippocampus, thalamus, and substantia nigra [212]. The density and distribution of tau depositions differ between age-matched controls and CTE, with CTE characterized by perivascular tau deposits localized at the depths of cortical sulci [74]. Experimental studies have further shown that brain homogenates derived from TBI mice, when inoculated into naïve animals, induce widespread tau pathology, synaptic loss, and persistent memory deficits, supporting a prion-like transmissible mechanism of tau propagation [213]. Evidence also suggests that temporality is an important factor since P-tau deposition emerges years to decades after injury; however, in TBI survivors, it often appears at a younger age than in controls, suggesting that trauma accelerates disease onset [214]. In wild-type mice, severe TBI initiates progressive tau accumulation, initially localized to the injured hemisphere and later spreading to contralateral regions over 12 months [213,215]. Tau protein aggregates in these models resemble neurofibrillary tangles and glial inclusions, frequently with perivascular localization [216]. These results establish the view that trauma-induced tau pathology is self-propagating, with neuroinflammation creating an environment for amplification and spread.

At the mechanistic level, microglial activation has a crucial impact. Activated microglia release pro-inflammatory cytokines, namely IL-1β, IL-6, and tumor necrosis factor (TNF)-α, which exacerbate tau hyperphosphorylation through kinase-dependent cascades [217]. In vivo PET studies show sustained glial reactivity in athletes with repetitive concussion [10]. These inflammatory responses correlate with subsequent neurodegeneration. Another study reported a threefold increase in IL-1α–positive microglia after head injury, which correlated with β-amyloid precursor protein-positive neurites (R = 0.78, *p* < 0.05), indicating that inflammation directly promotes neurotoxic protein accumulation [218].

The inflammasome pathway further links inflammation with tau pathology. NOD-, LRR-, and pyrin domain-containing protein 3 (NLRP3) inflammasome was shown to regulate pathological tau accumulation, with its prolonged activation driving IL-1β release and caspase-mediated pyroptosis [219]. Experimental interventions targeting this pathway, such as inhibiting AKT/nuclear factor kappa-light-chain-enhancer of activated B cells (NFκB)/NLRP3 signaling, demonstrated a potential therapeutic role in attenuating microglial-mediated injury [220]. In addition, pericytes act as amplifiers of inflammation since friend leukemia integration 1 (Fli-1) after TBI increased pericyte apoptosis, blood–brain barrier (BBB) disruption, and pro-inflammatory cytokine release, exacerbating microglial activation [221]. Repetitive injuries are also associated with more persistent and severe inflammation than single injuries, confirming the neuropathological progression of CTE [222].

#### 2.5.5. Evidence from Animal Models Linking TBI to Neurodegeneration

Preclinical models have provided important mechanistic insights into the way that TBI promotes chronic neurodegeneration, highlighting the combination of immune responses, neuronal death pathways, amyloid and tau pathology, and biomarker signatures. Ayerra et al. (2025) [223] demonstrated that adaptive immunity contributes substantially to the long-term progression of TBI. By using T-cell-deficient mice (T cell receptor beta (TCRβ)−/−δ−/−) subjected to controlled cortical impact, it was found that the absence of T-cells heavily reduced monocyte infiltration, attenuated microglial proliferation, and enhanced an anti-inflammatory phenotype in myeloid cells [223]. At the cellular level, evidence indicates that zipper-interacting protein kinase (ZIPK) has been implicated in mediating neuronal apoptotic pathways following TBI. In both controlled cortical impact models and in vitro neuronal injury cases, ZIPK was upregulated in neurons, reaching a peak at two days post-injury and persisting for a week [224]. Partial deletion of ZIPK (Zipk+/− mice) reduced neuronal death, while knockdown experiments in SH-SY5Y cells confirmed a neuroprotective effect under oxidative stress. Biomarkers from animal models have also increasingly been used since they align with preclinical findings in human TBI. In a systematic review of 74 studies, Lisi et al. (2025) [225] reported that glial fibrillary acidic protein (GFAP), ubiquitin terminal hydrolase L1 (UCH-L1), neurofilament light (NfL), and tau isoforms show specific temporal behavior post-injury. GFAP and UCH-L1 rose sharply within 24 h, while NfL elevations persisted for months, demonstrating chronic axonal injury, similarly seen in humans. However, tau biomarkers rose progressively, peaking over weeks, reflecting a long-term neurodegenerative process.

Amyloid pathology has also been researched robustly in animal models, linking DAI and impaired axonal transport to amyloid-beta accumulation. Rodent and swine studies showed that intra-axonal amyloid precursor protein (APP) accumulates as an early and consistent response to TBI, with amyloid-beta formation presenting more slowly. In APP-transgenic mouse models, TBI induced elevations in Aβ levels, peaking within hours; on the other hand, in swine rotational acceleration models, intra-axonal Aβ and diffuse plaque deposition were observed within 3–10 days, persisting for months [203,226]. Additionally, ApoE genotype studies indicated that APOε4-expresssing mice showed earlier and more pronounced Aβ deposition than those expressing ApoE3 [227]. Uryu et al. (2002) [228] demonstrated that repetitive but not single mild TBI in Tg2576 mice accelerated Aβ deposition, lipid peroxidation, and cognitive dysfunction. At 16 weeks post-trauma, repetitive TBI animals exhibited higher isoprostane levels, increased Aβ plaques, and memory impairment, whereas wild-type mice did not develop amyloidosis.

As a result, animal model evidence supports a temporal sequence, extending from acute biomarker release to chronic Aβ and tau accumulation, thereby providing biological plausibility mediated by immune, apoptotic, and metabolic pathways.

### 2.6. Causal Relationship Between TBI and AD

The link between traumatic brain injury (TBI) and Alzheimer’s disease (AD) is increasingly being conceptualized within a causal relationship, supported by epidemiological evidence, biological plausibility, and temporality. Evidence suggests that TBI, when moderate to severe, may accelerate processes leading to AD.

#### 2.6.1. Epidemiological Evidence Linking TBI to Dementia and Alzheimer’s Disease

Epidemiological studies consistently indicate that a history of a previous traumatic brain injury (TBI) confers an increased risk of late-life neurodegeneration, though the association with Alzheimer’s disease remains uncertain. A comprehensive population-based analysis found that individuals with a history of TBI exhibited a significantly elevated risk of Alzheimer’s disease-related neurodegeneration compared to age- and sex-adjusted referents. A large-scale cohort study of 1418 confirmed TBI cases and 2836 matched controls found that TBI was associated with 32% increased risk of Alzheimer’s disease and related dementias (HR = 1.32, 95% CI: 1.11–1.58, *p* = 0.002). Risk was significant for “probable” (HR = 1.42, 95% CI: 1.05–1.92) and “possible” (HR = 1.29, 95% CI: 1.02–1.62) TBI, but not for “definite” TBI (HR = 1.22, 95% CI: 0.68–2.18) [229]. Study populations are heterogeneous, ranging from general population cohorts to at-risk groups such as active-duty military personnel, veterans, and professional rugby and football athletes, most of whom were males and exposed to repetitive head impacts (RHIs). Epidemiology shows that rugby players are more likely to manifest a neurodegenerative disease compared with matched community controls [230].

Mild symptomatic TBI is the most common injury in rugby, and repetitive head impact, which may be symptomatic, has been linked to poorer cognitive function, mental health issues, and poor sleep quality [231]. A systematic review and meta-analysis by Snowden et al. (2020) [232] pooled data from 21 studies and reported that individuals with a history of mTBI had nearly twice the risk of developing dementia compared to those without prior head trauma (OR = 1.96, 95% CI 1.698–2.263).

Similarly, an umbrella systematic review confirmed that TBI across all severities is significantly associated with dementia risk (OR = 1.81, 95% Cl: 1.53–2.14). Stratification by severity indicated that both mild (OR = 1.96, 95% CI: 1.70–2.26) and moderate-to-severe TBI (OR = 1.95, 95% CI: 1.55–2.45) carried comparable increased risks. However, the association between TBI and Alzheimer’s disease was weaker and inconsistent across studies, with some analyses reporting modest associations (RR = 1.18, 95% CI: 1.11–1.25) and others detecting no significant relationship (OR = 1.02, 95% CI: 0.91–1.15) [233]. Barnes et al. (2014) [234] found that older U.S. Veterans with TBI history had a 60% increased risk of dementia (HR = 1.57, 95% CI 1.35–1.83), with risk persisting after a mean follow-up of 9 years. Graham et al. (2025) [184] demonstrated biomarker evidence of neurodegeneration in mid-life former rugby players, while Saltiel et al. (2024) [235] ighlighted the frequent coexistence of mixed proteinopathies (amyloid-β, tau, TDP-43, α-synuclein) in post-mortem tissue from individuals exposed to RHI. Post-mortem studies demonstrated a wide range of proteinopathies after RHI/TBI, including Aβ, TDP-43, alpha-synuclein, and tau, in addition to other neuropathologies such as white matter rarefaction and vascular injury [184]. The TRACK-TBI longitudinal study further analyzed the chronic outcomes by showing that the mTBI and mild severity TBI group survivors presented higher rates of cognitive, psychiatric, and functional decline observed years after initial injury, when compared with orthopedic trauma controls (OTCs) [236]. Hammond et al. (2004) [237] showed progressive communicational, cognitive, and social functioning problems between 1 and 5 years following the injury.

However, risk prediction, exposure assessment, and follow-up remain a limitation as indicated by Hanrahan et al. (2023) [238], who stated that 40% of studies reported no significant association, while 60% found increased risk or earlier dementia onset. Notably, acute severe TBI has been associated with widespread amyloid plaque pathology, but this is considered distinct from Alzheimer’s pathology [239]. Dams O’Connor et al. 2013 [240], who followed 4200 dementia-free individuals aged 65 years or older who were evaluated biannually, with a mean follow-up of 7.4 years, and a history of TBI with loss of consciousness, did not find an increase in the risk for development of dementia.

#### 2.6.2. Relationship Temporality

One of Bradford Hill’s requirements for causation is temporality, namely that the exposure precedes the outcome. The temporal association linking TBI or RHI and the later emergence of neurodegenerative disease has been demonstrated across human and preclinical studies. Epidemiological cohorts suggest that exposure to TBI, especially when accompanied by loss of consciousness (LOC), predicts later-life dementia [241]. The MIRAGE study, a large family-based case–control investigation involving more than 16,000 relatives, reported that head injury with LOC was associated with an odds ratio (OR) of up to 9.9 for Alzheimer’s disease (AD), a risk that persisted for decades after the initial trauma [242]. Neuropathological and imaging studies further enhance this temporal association by demonstrating progressive structural and functional brain alterations that persist long after injury. Longitudinal morphometric analysis from the DIAGNOSE CTE research in former American football players showed cortical thinning of the hippocampus, amygdala, and frontal and temporal cortices decades after exposure, with greater atrophy in professional athletes compared to amateurs, highlighting a dose–response relationship between RHI and neurodegeneration [243]. Similar findings were observed in a study of 185 athletes, in which longer duration in athletic career predicted gyral thinning and neuronal loss, suggesting a cumulative temporal burden of head impact exposures that leads to gyral vulnerability. Preclinical data focused on the mechanistic pathway in CD1 mice showed that a single severe TBI induced sustained brain atrophy, gliosis, reduced cerebral perfusion, and accumulation of beta-amyloid and hyperphosphorylated tau, with cognitive decline presenting over an 11-month follow-up period [244]. These data confirm that the neurodegenerative process evolves slowly after the inciting trauma, proving that temporality is a key element in this cascade. However, some prospective studies have not identified accelerated cognitive decline among TBI survivors when compared to controls. Long-term follow-up for over a decade, incorporating amyloid and tau PET imaging in chronic TBI survivors, revealed no elevation of β-amyloid or tau compared to healthy individuals [245]. These data showcase heterogeneity in post-TBI trajectory; nevertheless, when considered collectively, epidemiological and neuroimaging evidence support a temporal sequence establishing that injury precedes and can initiate neurodegenerative change.

#### 2.6.3. Biological Plausibility

Biological plausibility constitutes an important criterion within Bradford Hill’s framework, emphasizing the necessity for further mechanistic and translational analyses to clarify underlying causal pathways [246]. Evidence that stems from pathological, imaging, and preclinical studies indicates strong biological plausibility for a causal relationship between TBI and dementia. Human post-mortem studies of individuals with prior head trauma reveal a polypathology mechanism, including amyloid-β plaques, hyperphosphorylated tau, TDP-43 inclusions, and Lewy bodies, which are consistent with different neurodegenerative processes rather than a single disease pathway [247]. In addition, former athletes with white matter rarefaction and hippocampal sclerosis were independently associated with dementia, suggesting that axonal and vascular injury are central mechanisms irrespective of tau pathology [248]. Oxidative stress and inflammatory cascades have also been investigated from cellular and molecular perspectives. TBI-induced oxidative stress accelerates tau phosphorylation and amyloid-β aggregation, two core hallmarks of AD [249]. Chronic neuroinflammation mediated through NLRP3 inflammasome activation leads to sustained release of pro-inflammatory cytokines and neuronal death, processes also shared with AD and CTE [250,251]. Neuroimaging findings suggest in vivo evidence of these mechanisms. Enlarged perivascular spaces in mTBI survivors demonstrated impaired lymphatic clearance and the accumulation of toxic proteins [252]. Axonal injury within the anterior internal capsulae was identified as a predictor of sleep disturbance and memory decline, indicating tract-specific vulnerability [253]. Smaller hippocampal volumes after TBI were also reported, particularly within the CA1 region, with a stronger association in older adults, highlighting how injury interacts with aging processes to exacerbate neurodegeneration [254]. In conclusion, TBI initiates a cascade of vascular, axonal, inflammatory, and proteinopathic processes that lead to progressive neurodegenerative disease. Table 1 provides a comparative overview of the initiating events, neuroanatomical distribution, pathology and imaging characteristics distinguishing post-traumatic TBI, CTE and AD.

### 2.7. Therapeutic Interventions

**Figure 1:**
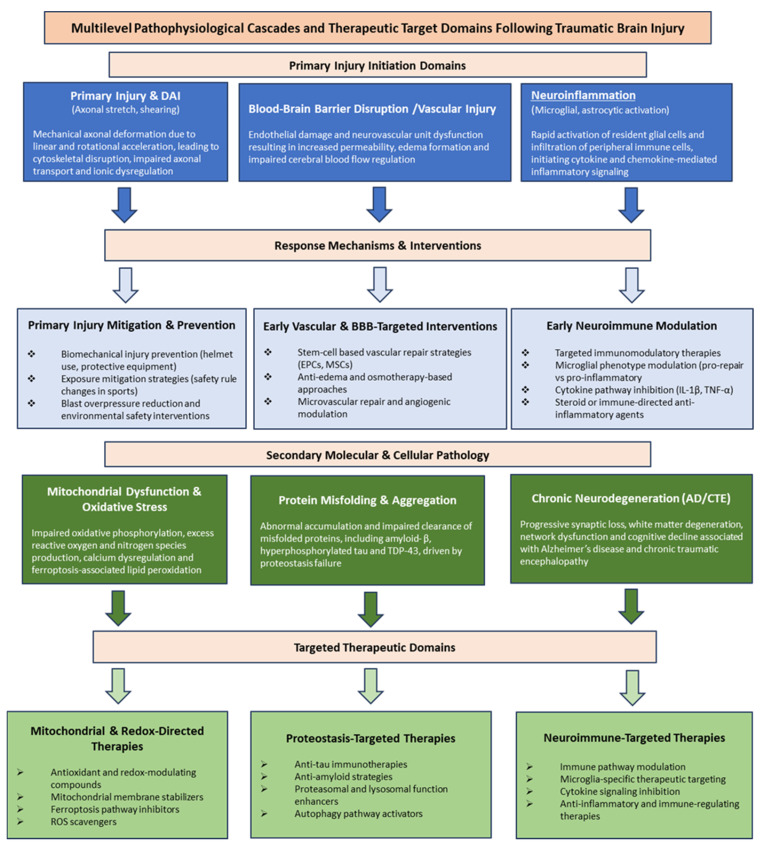
Multilevel pathophysiological cascades and therapeutic target domains following TBI. The figure illustrates the hierarchical organization of injury mechanisms, secondary biological processes, and therapeutic target domains following TBI. Primary injury domains include mechanical axonal injury and DAI, BBB disruption with neurovascular unit dysfunction, and early neuroinflammatory activation involving microglia and astrocytes. These processes trigger coordinated response mechanisms and intervention strategies aimed at mitigating injury severity, preserving vascular integrity, and modulating early immune response. Secondary molecular and cellular pathologies such as mitochondrial dysfunction and oxidative stress, protein misfolding and aggregation, and progressive neurodegeneration follow. The lower tier highlights targeted therapeutic domains aligned with these pathological pathways.

Traumatic brain injury (TBI) remains a complex neuropathological condition with disease-specific therapy yet to be established. The available literature showed a range of experimental and preclinical approaches with the main aim of mitigating the pathological mechanisms of TBI, such as neuronal degeneration, excitotoxicity, blood–brain barrier (BBB) disruption, mitochondrial dysfunction, and oxidative stress. Current therapeutic solutions could be further categorized into acute care and neuroprotection, stem-cell-based therapies for BBB repair, mitochondrial stabilization, transcriptional and hormonal modulation, and immunomodulatory approaches aimed at mitigating neuroinflammation. Figure 1 summarizes the proposed pathophysiological mechanisms and therapeutic models of action following TBI.

#### 2.7.1. Stem Cell-Based Approaches for BBB and Neural Repair

Several preclinical studies demonstrated the capacity of stem cell-based therapies to restore BBB integrity and mitigate neurodegenerative processes. There are different classes of stem cells, namely neural stem cells (NSCs), endothelial progenitor cells (EPCs), hematopoietic stem cells (HSCs), and mesenchymal stem cells (MSCs), which can preserve and promote BBB repair [255,256]. Neural stem cells (NSCs), which self-renew and differentiate into neurons, astrocytes, and oligodendrocytes, demonstrated potential to establish disrupted neural circuits at the site of injury. Preclinical studies by Xia et al. 2025 [256] revealed that transplantation of neural stem cells or premature neurons to the injury site introduces new functional neurons, potentially re-establishing neural circuits and promoting brain tissue repair. They engineered NSCs with micro-patches via microcontact printing, enabling sustained release of retinoic acid, which increased neuronal differentiation from 28% to 54% in vitro without affecting the viability [256]. Intranasal administration of human NSCs restored pericyte populations and reduced amyloid beta pathology in APP/Presenilin-1 protein (PS1) transgenic mice, suggesting translational potential for Alzheimer’s disease [257]. EPCs are bone marrow-derived cells that can migrate to injury sites to proliferate and differentiate into mature endothelial cells, enhancing angiogenesis and vasculogenesis [258]. In TBI models, intracerebroventricular transplantation of EPCs upregulated tight junction proteins, reduced BBB leakage due to disruption, and improved neurological outcomes after TBI [259]. Complementary findings in APP/PS1 mice showed that stereotactic injection of EPCs into the hippocampus enhanced BBB integrity and facilitated Aβ clearance, ultimately improving cognitive outcomes [260]. HSCs are undifferentiated multipotent progenitors for both myeloid and lymphoid lineages, which have also been implicated in BBB repair and modulation of neuroinflammation [261]. In a recent study, Mishra et al. 2023 [262] demonstrated that transplantation of HSCs from wild-type donors into 5x FAD mice reduced amyloid plaque deposition, mitigated neuroinflammation, reinforced BBB integrity, and improved cognitive outcomes. MSCs are multipotent adult stroma stem cells derived from the bone marrow and have been shown to act primarily through paracrine mechanisms [263]. Intravenous administration of MSCs preserved endothelial adherent and tight junctions and protected against BBB dysfunction following TBI [264]. These studies underscore the importance of the therapeutic potential of different stem cell classes in addressing both vascular and neuronal dysfunction after TBI and AD-related pathology. To date, these findings remain preclinical, and no large-scale clinical trials have established efficacy or safety in human TBI patients.

#### 2.7.2. Natural Compounds as Neuroprotective Agents

Secondary injury cascades in TBI involve oxidative stress, excitotoxicity, and BBB disruption. Brooshgahalan (2024) [265] reported that fucoidan, a sulfated polysaccharide extracted from seaweed, exerts potent neuroprotective effects when administered acutely post-injury. It was shown to improve neurological scores, restore BBB integrity, and reduce oxidative damage, while preserving the hippocampal long-term potentiation, a synaptic mechanism essential for cognitive recovery, highlighting its potential role as an antioxidant compound in adjuvant therapy. Melatonin has also emerged as a promising neuroprotective agent. Across multiple preclinical studies, melatonin acts through various mechanisms, including modulation of caspase-dependent apoptosis, activation of the Nrf2 oxidative stress pathway, and stimulation of mitophagy [266,267,268]. More recent work by Gunara et al. 2025 [269] revealed an additional effect in reducing demyelination, suggesting a potential broader role in preserving glial and myelin integrity. These effects were dose-dependent, with optimal benefit observed at 10 mg/kg. Clinical validation of these compounds in TBI patients requires further optimal dosing, timing, and safety profiles to be established.

#### 2.7.3. Targeting Mitochondrial Dysfunction

Mitochondrial dysfunction was identified as a central driver of neuronal injury. Carteri et al.’s 2025 [270] study proposed that accumulated reactive oxygen species (ROS) within mitochondria contribute to neuronal damage [270]. Beyond pharmacological stabilization, mitochondrial transplantation was also investigated as a potential therapy for TBI, supporting its capacity to restore energy metabolism and prevent cell death [271]. The broader literature has also linked defective mitophagy and ROS accumulation to neurodegenerative diseases such as Alzheimer’s disease, Huntington’s disease (HD), and amyotrophic lateral sclerosis (ALS) [272,273]. As a result, these findings suggest that interventions aimed at mitochondrial stabilization could represent a therapeutic solution across TBI subtypes and subsequent neurodegeneration.

#### 2.7.4. Transcriptional and Hormonal Modulation

Transcriptomic analyses have highlighted the potential for reprogramming injured neurons. Alkalasi et al. 2025 [274] revealed that mild TBI activates the transcription factor Atf3, a stress-responsive transcription factor previously associated with peripheral nerve regeneration. Interestingly, cortical neurons exhibited transcriptional responses distinct from those observed in peripheral neurons, suggesting that targeted reprogramming of CNS transcriptional pathways may require targeted modulation to restore synaptic plasticity and functional recovery. Endocrine modulation has also been investigated as a therapeutic strategy. Zhang et al. 2024 [275] showed that thyroxine (T4) treatment resulted in restoring the disrupted astrocyte and microglia transcriptional networks following TBI, particularly in the hippocampus area. Normally, thyroid hormones such as thyroxine (T4) and triiodothyronine (T3) are important for physiological brain development and maturation [276]. This intervention not only promoted the acute injury repair but also reversed dysregulated metabolic and immune pathways. T4-regulated genes overlapped with human genome-wide association study (GWAS) loci for cognition and psychiatric disorders, providing translational importance. Other studies have also shown that post-injury administration of triiodothyronine (T3) improves motor and cognitive recovery, reduces lesion volume and neuroinflammation, and mediates the upregulation of neurotrophic factors such as brain-derived neurotrophic factor (BDNF) and glial cell line-derived neurotrophic factor (GDNF) [277,278].

#### 2.7.5. Immunomodulation and Neuroinflammation

Primary mechanical injury following TBI initiates a complex neuroinflammatory cascade involving cytokines and chemokines that organize the function of brain cells, such as microglia, astrocytes, and infiltration of peripheral immune cells [279]. While acute inflammation contributes to repair, chronic activation exacerbates secondary injury by producing pro-inflammatory cytokines, including tumor necrosis factor-alpha (TNF-α), interleukin 6 (IL-6), interleukin 1 beta (IL-1β), and interferon-gamma (IFN-γ) [280]. Inflammation has an equivocal role, being initially protective yet chronically maladaptive. Single TBI events may induce transient activation of repair-associated immune pathways, whereas repetitive TBI is linked to persistent neuroinflammatory signaling, synaptic dysfunction, and dysregulation of proteins (e.g., ApoA1, ApoE, Cox6a1, and Snca) [145]. These chronic inflammatory states are being increasingly linked to progressive neurodegenerative changes. Accordingly, immunomodulatory therapies targeting inflammation have been explored to mitigate chronic inflammation. Czaerpaniak et al. (2024) [281] investigated delayed administration (4–6 weeks post-injury) of low-dose interleukin-2 after mild TBI, which reversed chronic headache symptoms and restored cognitive performance. In parallel, monoclonal antibodies (mAbs) targeting inflammatory cytokines (IL-6, IL-1β, and TNF-α) and neurodegenerative proteins (Aβ, tau, α-synuclein) demonstrated efficacy in preclinical TBI models [282]. Kondo et al. 2015 [283] reported that antibodies targeting cis-phosphorylated tau reduced tau accumulation and restored neuronal function, supporting immunotherapy as a potential disease-modifying strategy. 

#### 2.7.6. Neurovascular-Linked Therapies

Neurovascular-linked therapies have emerged as a potential intervention based on the shared neurovascular hypothesis of TBI and Alzheimer’s disease. Drugs initially developed for cardiovascular intervention, namely telmisartan, simvastatin, dabigatran, and 3K3A-activated protein C (3K3A-APC), have shown anti-inflammatory, antioxidant, and neuroprotective effects in mitigating BBB disruption, proteinopathies, and microvascular injury in preclinical TBI and AD animal models [284].

Collectively, the experimental and preclinical studies show that traumatic brain injury could be characterized as a biologically modifiable condition at multiple levels, including blood–brain barrier integrity, mitochondrial function, transcriptional regulation, and neuronal immune signaling. Findings derived from in vitro studies and animal models indicate that interventions such as stem cell-based strategies, naturally derived neuroprotective agents, mitochondrial stabilization, and hormonal modulation can attenuate secondary injury mechanisms and further limit neurodegenerative progression, improving functional recovery. However, despite strong mechanistic support and reproducible benefits in controlled experimental settings, these approaches remain largely preclinical. These findings underscore both the therapeutic potential of targeting shared neurovascular and neuroinflammatory pathways in TBI and the critical need for clinical trials to enable the progression from experimental efficacy to clinical impact.

#### 2.7.7. Pharmacological Interventions in Acute and Chronic TBI

Unlike the preceding experimental approaches, the interventions discussed in this section represent therapies currently used or evaluated in human TBI patients, with evidence derived from clinical trials and practice guidelines. TBI management is planned according to the post-injury timeline. In the acute phase (0–3 h), interventions focus on hemostasis, intracranial pressure (ICP) control, seizure prophylaxis, and prevention of secondary injury cascades [285]. Tranexamic acid is administered within three hours to limit hemorrhagic expansion in mild to moderate TBI [286], while reversal of anticoagulants (Vitamin K, fresh frozen plasma [FFP], and direct oral anticoagulants [DOACs]) and correction of thrombocytopenia are important to prevent hemorrhage progression. Hyperosmolar agents such as mannitol and hypertonic saline are used to handle intracranial hypertension and cerebral edema [287]. For refractory ICP management and reduction in cerebral metabolism, sedatives such as barbiturates are used [288]. Anticonvulsants are used to prevent early post-traumatic seizures, and antipyretics are used to enhance cerebral oxygenation [289]. Investigational pharmacological therapies in this phase involve corticosteroids, citicoline, and progesterone, although clinical trials show mixed results [290]. The CRASH trial for corticosteroids demonstrated no improvement in the long-term outcomes after TBI, and citicoline failed to improve recovery in the COBRIT study, while progesterone showed efficacy in TBI models [291,292,293].

In the post-acute and chronic phases, focus is on neurobehavioral, cognitive, and neuropsychiatric support [290]. Selective serotonin reuptake inhibitors (SSRIs) are used to treat depression and attention deficits, while tricyclic antidepressants (TCAs) target mood and sleep disturbances [294,295].

## 3. Conclusions

Substantial progress has been made in linking traumatic brain injury (TBI) and diffuse axonal injury (DAI) to long-term neurodegenerative outcomes. However, several critical gaps still require focused investigation, necessary to prove causality, develop innovative therapeutic approaches, and refine strategies for early detection and prevention of Alzheimer’s disease and other dementias.

One major ongoing challenge lies not in the absence of appropriate experimental models but in the heterogeneity and translational limitations of existing preclinical TBI models. A wide range of injury models, including controlled cortical impact, blast exposure, rotational acceleration, and repetitive mild TBI, have been developed to reproduce focal and diffuse axonal injury with varying degrees of success.

To enhance translational relevance, future studies should incorporate broader biomechanical diversity, including combinations of focal and diffuse injury, repetitive exposure paradigms, and stratification by age and sex.

In parallel, there is a critical need to standardize and validate fluid and imaging biomarkers across cohorts and time periods. Current evidence focuses on GFAP, UCH-L1, NfL, S100B, and tau as chosen biomarkers. Nevertheless, longitudinal cohort studies with standardized sampling approaches are crucial to characterize acute, subacute, and chronic biomarker pathways and to determine whether these trajectories enhance the risk of Alzheimer’s pathology. Risk stratification by demographic and genetic factors is also required for future investigations. Age and sex both affect TBI outcomes, as well as modulate the vulnerability to progressive neurodegeneration. Women tend to present different biomarker profiles and recovery patterns compared to men, while older patients exhibit slower clearance of injury-related proteins and a higher risk of cognitive decline. The APOE ε4 allele, which is already established as a genetic risk factor for sporadic AD, has been proposed to exacerbate the impact of TBI on amyloid deposition and clinical progression. For this, large-scale, genotype-categorized studies will be necessary to fully clarify this interaction. Biomechanical studies highlight the need for more accurate modeling of brain tissue responses to rotational and linear acceleration, as current viscoelastic approximation does not fully resemble the human neuroanatomical complexity.

Therapeutically, intervention temporality plays a crucial role. As highlighted, TBI pathophysiology implicates acute excitotoxicity, subacute neuroinflammation, and delayed tauopathy, indicating the need for multiple therapeutic interventions. However, it appears essential to temporally combine therapies that target excitotoxic cascades and neurovascular unit stabilization, followed by the subacute modulation of inflammation and chronic strategies to reduce tau aggregation.

Ultimately, a multimodal framework that combines TBI models, standardized biomarkers, stratification by sex, age, and genotype, and temporally adapted therapeutic approaches is necessary. Consequently, we could resolve the major question of whether TBI increases the risk of Alzheimer-type pathology or whether it produces spatial vulnerability across brain regions.

In conclusion, traumatic brain injury represents not only an acute neurological event but also a potential initiator of chronic neurodegenerative processes, which overlap with Alzheimer’s disease and chronic traumatic encephalopathy. Across epidemiological, clinical, and biomechanical analyses, evidence suggests that TBI is associated with long-term cognitive decline, psychiatric comorbidities, and pathological hallmarks, namely amyloid-β deposition, tau hyperphosphorylation, and neuroinflammation. Nevertheless, the relationship remains complex, influenced by injury severity, frequency, anatomic location, age, sex, and genetic susceptibility. In sum, TBI plays a pivotal role at the intersection of acute injury and chronic neurodegeneration. For this reason, discovering the connection between preclinical studies, biomarker validation, and clinical interventions is important for enhanced understanding of TBI-related neurodegeneration in developing strategies for diagnosis, prevention, and appropriate treatment.

## Figures and Tables

**Table 1:** Comparison of post-traumatic neurodegeneration, chronic traumatic encephalopathy, and Alzheimer’s disease.

	Post-TBI Neurodegeneration	Chronic Traumatic Encephalopathy (CTE)	Alzheimer’s Disease (AD)
Initiating Events	Single or repetitive TBI across the severity spectrum (mild-severe), including blast exposure	Repetitive head impacts (RHI), often sub-concussive, associated with contact sports or military blast exposure	Multifactorial etiology involving advanced age, genetic susceptibility (APOε4, TREM2 variants)
Neuroanatomical Topography	Selective vulnerability of long association and white matter tracts, including corpus callosum, internal capsule, with diffuse involvement of deep and periventricular white matter networks	Predominant involvement of cortical sulcal depths, especially in frontal and temporal cortices, with a characteristic perivascular distribution, and extends to limbic structures and brainstem in advanced stages	Early involvement of medial temporal lobe structures (hippocampus and entorhinal cortex), followed by spread to temporoparietal association cortices
Predominant Pathology	Diffuse axonal injury and microvascular disruption. Chronic cases may demonstrate mixed proteinopathy and accumulation of tau and β- amyloid	Pathognomonic perivascular accumulation of hyperphosphorylated tau at sulcal depths, involving both neurons and astrocytes, accompanied by frequent TDP-43 co-pathology in the medial temporal lobe, diencephalon, and progressive degeneration of white matter	Accumulation of extracellular neuritic β-amyloid plaques and intracellular neurofibrillary tangles composed of hyperphosphorylated tau, beginning in the entorhinal cortex and hippocampus, and spreading to temporoparietal and frontal association
Imaging	White matter atrophy, reduced fractional anisotropy on diffusion MRI, and microbleeds on susceptibility-weighted imaging, patterns of cortical atrophy	Regional cortical thinning and sulcal atrophy have been described, alongside tau PET signal in characteristic sulcal and perivascular regions, while standard MRI may be non-specific	Hippocampal and temporoparietal cortical atrophy on structural MRI, positive amyloid and tau PET, imaging consistent with established AD biomarker patterns

These entities share overlapping features such as tau pathology and cortical atrophy; however, they differ in the primary initiating event, the anatomical distribution of the lesion, and the imaging findings. Post-traumatic neurodegeneration is characterized by diffuse axonal and microvascular injury, CTE by perivascular sulcal tau pathology following the repetitive head impacts, and Alzheimer’s disease by hippocampal and temporoparietal amyloid and tau deposition.

## Data Availability

No new data were created or analyzed in this study.

## Abbreviations

The following abbreviations are used in this manuscript:

3K3A-APC	3K3A-activated protein C
AD	Alzheimer’s disease
ADAM10	A disintegrin and metalloproteinase domain-containing protein 10
ALS	Amyotrophic lateral sclerosis
AMPA	α-amino-3-hydroxy-5-methyl-4-isoxazolepropionic acid
ApoE	Apolipoprotein E
APP	Amyloid precursor protein
ATP	Adenosine triphosphate
Aβ	Amyloid-beta
BACE1	β-secretase
BAG3	BCL2-associated athanogene 3
BBB	Blood–brain barrier
BDNF	Brain-derived neurotrophic factor
Brain-PAG	Brain-predicted age gaps
bvFTD	Behavioral variant frontotemporal dementia
Cav-1	Caveolin-1
CBF	Cerebral blood flow
CBI-M	Clinical, Biomarker, Imaging Modifiers
CCL2	C-C motif chemokine ligand 2
CISD1	CDGSH iron–sulfur domain-containing protein 1
CNS	Central nervous system
CSF	Cerebrospinal fluid
CTE	Chronic traumatic encephalopathy
CypA	Cyclophilin A
DAI	Diffuse axonal injury
DHODH	Dihydroorotate dehydrogenase
DOACs	Direct oral anticoagulants
DTI	Diffusion tensor imaging
EPCs	Endothelial progenitor cells
FA	Fractional anisotropy
FFP	Fresh frozen plasma
FLAIR	Fluid-attenuated inversion recovery
Fli-1	Friend leukemia integration 1
FTLD	Frontotemporal lobar degeneration
GCS	Glasgow Coma Scale
GDNF	Glial cell line-derived neurotrophic factor
GFAP	Glial fibrillary acidic protein
GWAS	Genome-wide association studies
HD	Huntington’s disease
HSCs	Hematopoietic stem cells
ICAM	Intercellular adhesion molecule
ICP	Intracranial pressure
IFN-γ	Interferon-gamma
IL	Interleukin
LOC	Loss of consciousness
LRP1	Low-density lipoprotein receptor-related protein 1
mAbs	Monoclonal antibodies
MAG	Myelin-associated glycoprotein
MeSH	Medical Subject Headings
Mfsd2a	Major facilitator superfamily domain-containing protein 2A
MMP-9	Matrix metalloproteinase-9
MND	Motor neuron disease
MRI	Magnetic resonance imaging
MSCs	Mesenchymal stem cells
mTOR	Mechanistic (mammalian) target of rapamycin
NfL	Neurofilament light chain
NFTs	Neurofibrillary tangles
NFκB	Nuclear factor kappa-light-chain-enhancer of activated B cells
NLRP3	NOD-, LRR-, and pyrin domain-containing protein 3
NMDA	N-methyl-D-aspartate
NSCs	Neural stem cells
NSE	Neuron-specific enolase
NVU	Neurovascular unit
OR	Odds ratio
OTCs	Orthopedic trauma controls
OXPHOS	Oxidative phosphorylation
PCC	Population-Concept-Context
PD	Parkinson’s disease
PET	Positron emission tomography
PS1	Presenilin-1 protein
PSEN1	Presenilin-1 gene
P-tau	Phosphorylated tau
RHI	Repetitive head impacts
RNS	Reactive nitrogen species
ROS	Reactive oxygen species
RR	Risk ratio
Shh	Sonic hedgehog
SNAP-25	Synaptosomal-associated protein 25
SRC	Sports-related concussion
SSRIs	Selective serotonin reuptake inhibitors
sTREM2	Soluble triggering receptor expressed on myeloid cells 2
T3	Triiodothyronine
T4	Thyroxine
TBI	Traumatic brain injury
TBI-ND	Traumatic brain injury-related neurodegeneration
TCAs	Tricyclic antidepressants
TCR	T-cell receptor
TDP-43	Transactive response DNA-binding protein 43
TES	Traumatic encephalopathy syndrome
TGF-β	Transforming growth factor beta
TNF	Tumor necrosis factor
TOMM40	Translocase of outer mitochondrial membrane 40
T-tau	Total tau
UCH-L1	Ubiquitin carboxy-terminal hydrolase L1
VaD	Vascular dementia
VCAM-1	Vascular cell adhesion molecule-1
VEGF	Vascular endothelial growth factor
VILIP-1	Visinin-like protein 1
VSMCs	Vascular smooth muscle cells
Wnt	Wingless/Integrated
ZIPK	Zipper-interacting protein kinase
α-SMA	Alpha-smooth muscle actin
β-CTF	Beta C-terminal fragment of APP
